# Surface thiolation of silicon for antifouling application

**DOI:** 10.1186/s13065-018-0385-6

**Published:** 2018-02-07

**Authors:** Xiaoning Zhang, Pei Gao, Valerie Hollimon, DaShan Brodus, Arion Johnson, Hongmei Hu

**Affiliations:** 1 College of Biotechnology, Southwest University, Chongqing, 400715 China; 2 Department of Chemistry, Eastern Kentucky University, 521 Lancaster Ave, Richmond, KY 40475 USA; 3 Department of Mathematics, Sciences and Technology, Paine College, Augusta, GA 30901 USA; 4 Key Laboratory of Mariculture and Enhancement of Zhejiang Province, Marine Fishery Institute of Zhejiang Province, Zhoushan, 316021 China

**Keywords:** Silicon substrate, Surface thiolation, PFDT, Antifouling

## Abstract

Thiol groups grafted silicon surface was prepared as previously described. 1*H*,1*H*,2*H*,2*H*-perfluorodecanethiol (PFDT) molecules were then immobilized on such a surface through disulfide bonds formation. To investigate the contribution of PFDT coating to antifouling, the adhesion behaviors of *Botryococcus braunii* (*B. braunii*) and *Escherichia coli* (*E. coli*) were studied through biofouling assays in the laboratory. The representative microscope images suggest reduced *B. braunii* and *E. coli* accumulation densities on PFDT integrated silicon substrate. However, the antifouling performance of PFDT integrated silicon substrate decreased over time. By incubating the aged substrate in 10 mM TCEP·HCl solution for 1 h, the fouled PFDT coating could be removed as the disulfide bonds were cleaved, resulting in reduced absorption of algal cells and exposure of non-fouled silicon substrate surface. Our results indicate that the thiol-terminated substrate can be potentially useful for restoring the fouled surface, as well as maximizing the effective usage of the substrate.

**Electronic supplementary material:**

The online version of this article (10.1186/s13065-018-0385-6) contains supplementary material, which is available to authorized users.

## Introduction

Biofouling is a complex process that involves living organisms and cells probing and attaching to surfaces. Biofouling is a big challenge for the biomedical industry because biofilms form easily on surfaces such as door handles, surgical equipment, and many other medical devices and could increase the spread of disease in humans. Data have shown that an estimated 1.7 million infections are caused from healthcare-associated infections annually [1]. In addition, because the growth of marine organisms on ship hulls can cause a drag force, biofouling can also result in decreased fuel efficiency and increased fuel consumption [2–6].

One strategy to reduce biofouling adsorption is to passivate the substrate through the coupling of antifouling molecules such as poly(ethylene glycol) [7] or poly(ethylene glycol) dimethacrylate [8]. Various works in surface modification for antifouling purposes have been summarized and reported [9, 10]. An important challenge in the field of antifouling is that an antifouling coating does not last forever; it becomes less effective as it ages. It also has been brought to our attention that once the deposition of foulants has taken place, the surface modification no longer effectively prevents fouling, which is understandable considering that the effect of solute/coating interaction is severely reduced once a layer of deposited foulants is formed [10]. Therefore, once the fouling layer is formed, the old antifouling coating needs to be removed, and a new antifouling coating needs to be applied. One way of removing antifouling coating is by scraping, a time-consuming process that might damage the surface. We must therefore explore a method by which to remove the fouled coating easily.

Silicon materials are integral parts of our daily lives and have widespread applications in healthcare and manufacturing due to silicon’s unique material properties, including high flexibility, chemical and thermal stability, and ease of fabrication [9]. In addition, silicon materials are mechanically and chemically resilient-able to resist wear in aqueous and organic environments-and display good electrical properties. Therefore, in this study, silicon substrate was selected as a model. Previously, we had developed a technique that allowed us to coat thiol-terminated silicon substrate with PFDT molecules through disulfide bonds. Here, the antifouling property of the PFDT-coated silicon substrate was tested by aging the substrate in *Escherichia coli* (*E. coli*) and *Botryococcus braunii* (*B. braunii*) cultures respectively. A large amount of *B. braunii* colonies were found anchored on the substrate in a 30-day immersion test. However, by applying a reducing agent, the disulfide bonds could be cleaved and the fouled coating could be removed, therefore exposing a non-fouled silicon substrate.

## Experimental

### Chemicals and materials

#### Chemicals and materials for thiolation

Anhydrous *N*,*N*-dimethylformamide (DMF; ≥ 99.8%) was purchased from Fisher Scientific (United States). Anhydrous benzene (≥ 98%), anhydrous alcohol (≤ 0.005% water), sodium hydrosulfide hydrate (pure), 1*H*,1*H*,2*H*,2*H*-perfluorodecanethiol (PFDT; 97%), tetrabutylammonium iodide (98%), benzoyl peroxide (≥ 98%), phosphorus chloride (≥ 98%), sulfuric acid (95–98%), hydrogen peroxide (30%, mass fraction), tris(2-carboxyethyl) phosphine hydrochloride (TCEP·HCl; ≥ 98%), and ultraflat silicon (111) wafers (N-type) were purchased from Sigma-Aldrich (United States).

#### Chemicals and materials for cultivation of *B. braunii* and *E. coli*

Calcium chloride dihydrate (≥ 99.0%), magnesium sulfate heptahydrate (≥ 98%), potassium phosphate dibasic (≥ 98%), potassium phosphate monobasic (≥ 99.0%), and Luria–Bertani (LB) broth were purchased from Sigma-Aldrich (United States). Sodium nitrate (≥ 99.0%) and sodium chloride (≥ 99.0%) were purchased from Fisher Scientific (United States). The system used for cultivating *B. braunii* is equipped with a Tetra Whisper aquarium air pump (United States) to introduce air bubbles.

### Synthesis of thiolated silicon substrate

The thiol-terminated silicon substrate was prepared as previously described [11]. Briefly, silicon wafers were cut into 1 cm × 1 cm pieces. The wafers were cleaned with Piranha solution and were hydrogenated in NH_4_F/HF(aq) solution at room temperature. The substrates were then chlorinated in a saturated solution of PCl_5_ with benzyl peroxide in anhydrous benzene. Following the surface chlorination, the chlorinated substrates were placed in a NaSH DMF solution for surface thiolation.

### Preparation of PFDT modified silicon substrate

**Fig. 1:**
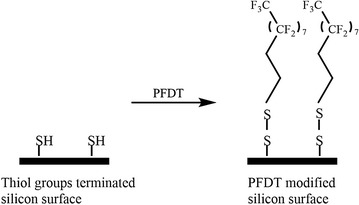
Schematic diagram for PFDT molecules modified silicon surface preparation

To prepare the PFDT modified silicon surface, the thiol-terminated substrate was submerged in 100 mmol L^−1^ PFDT anhydrous ethanol solution for 2 h immediately after the silicon surface thiolation process. Through the formation of the disulfide bonds, PFDT molecules are able to covalently bind onto the thiol-terminated silicon substrate as shown in Fig. 1. The prepared samples were then rinsed thoroughly with anhydrous ethanol, followed by sonicating in anhydrous ethanol for 5 min. This process was repeated for several times to remove physically adsorbed PFDT molecules from sample surface. The samples were then dried with a stream of nitrogen. The attachment of PFDT molecules onto thiol-terminated silicon substrate was approved by X-ray photoelectron spectroscopy (XPS) in which a strong F 1*s* peak was observed (Additional file 1: Figure S1).

### Surface characterization

The surface chemical composition of the modified silicon substrates were characterized by a Kratos Axis Ultra DLD XPS under an ultrahigh vacuum system at a base pressure of 1.33 × 10^−7^ Pa and equipped with a monochromatic Al K alpha source. Survey spectra were obtained at a 1 eV resolution, and high-resolution spectra were obtained at a 0.1 eV resolution. All spectral analysis was performed with the Kratos analytical software package (Vision 2.2.10 Rev 4).

Contact angle measurements (static angles) were conducted at 25 °C with 18 MΩ cm deionized (DI) water using a homemade experimental setup [12]. The droplet size used in contact angle measurements was 8 µL so that there was no influence of gravity on contact angle measurement [13]. Water contact angles were measured from four different positions on the surface. DI water was obtained from a Millipore Direct-Q 3 water purification system.

Light micrographs of *B. braunii* and methylene blue-stained *E. coli* were acquired by using a Nikon Eclipse 80i microscope (*E. coli* cannot be seen without staining through a microscope).

### Cultivation of algae

*Botryococcus braunii* was obtained from the Culture Collection of Algae at the University of Texas at Austin. *B. braunii* was selected as a model algae cell for this study because *B. braunii* is a green microalgae widely found in temperate or tropical lakes and estuaries. The algae cells were cultivated for a period of 14 days, which is when the *B. braunii* population reached a relatively stable phase in Bristol medium (2.94 mM NaNO_3_, 0.17 mM CaCl_2_·2H_2_O, 0.3 mM MgSO_4_·7H_2_O, 0.43 mM K_2_HPO_4_, 1.29 mM KH_2_PO_4_, and 0.43 mM NaCl) before the antifouling assay. Cultures were grown at 19 ± 2 °C with a 16 light/8 dark photoperiod. Lighting was supplied by a combination of warm and cold fluorescent tubes giving a luminance range of between 2200 and 2800 Lux. Continuous airflow was bubbled through the culture with a speed of 0.1 vvm, and pure carbon dioxide was supplemented to supply the carbon source every 48 h. The in vivo absorption of the culture medium containing algal cells in each flask was monitored each day via UV–Vis spectrophotometer 2450 (Shimadzu) at 660 nm (Additional file 1: Figure S2).

### Cultivation of *E. coli*

The best characterized *E. coli* strain, K-12 (American Type Culture Collection ATCC 25404, wild type), was provided by Dr. Yinan Wei of the University of Kentucky, Department of Chemistry, as a gift for this research. *E. coli* was maintained at 250 mL of Luria–Bertani (LB) broth at 37 °C, with shaking at 200 rpm for 12 h until the OD600 value was approximately 0.6. *E. coli* was used as a model organism because *E. coli* is a bacterium commonly found in the environment and is most widely studied.

## Results and discussion

**Fig. 2:**
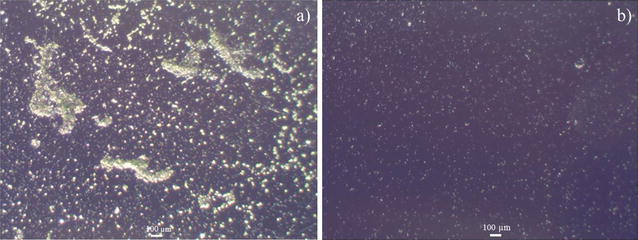
Representative microscope images of Piranha solution cleaned (**a**) and PFDT molecules modified silicon surface (**b**) after immersion test in *B. braunii* culture for 1 week. The population of *B. braunii* cells attached on the Piranha solution cleaned silicon surface is much higher than that on the PFDT molecules modified silicon surface. We can therefore conclude that PFDT coated silicon surface possesses fouling resistant properties regarding *B. braunii*

To evaluate the antifouling performance of PFDT modified silicon substrate, we immersed the PFDT-coated silicon wafer and Piranha solution (one part 98% H_2_SO_4_ and two parts 30% hydrogen peroxide) cleaned silicon wafer in a *B. braunii* culture. As shown in Fig. 2, after culturing for 1 week, there were large amount of algal cells adhered on the Piranha solution cleaned silicon wafer (Fig. 2a), whereas fewer algal cells adhered on the PFDT modified silicon wafer (Fig. 2b). *B. braunii* cells on Piranha solution cleaned silicon substrate grew in small groups, and some of them formed clusters (Fig. 2a). Our observations are in agreement with the previous studies regarding the typical stages of bio-fouling development that bioorganisms can multiply locally and then assemble to form microcolonies [14, 15]. The obvious reduction in the number of algal cells adhered to PFDT modified silicon substrate (Fig. 2b) indicates such a surface has resistance to the adhesion of algal cells, and it is not “algal friendly”. This result is consistent with other published studies involving the fluorination of substrates being applied to minimize microbial adhesion [16–18].

**Fig. 3:**
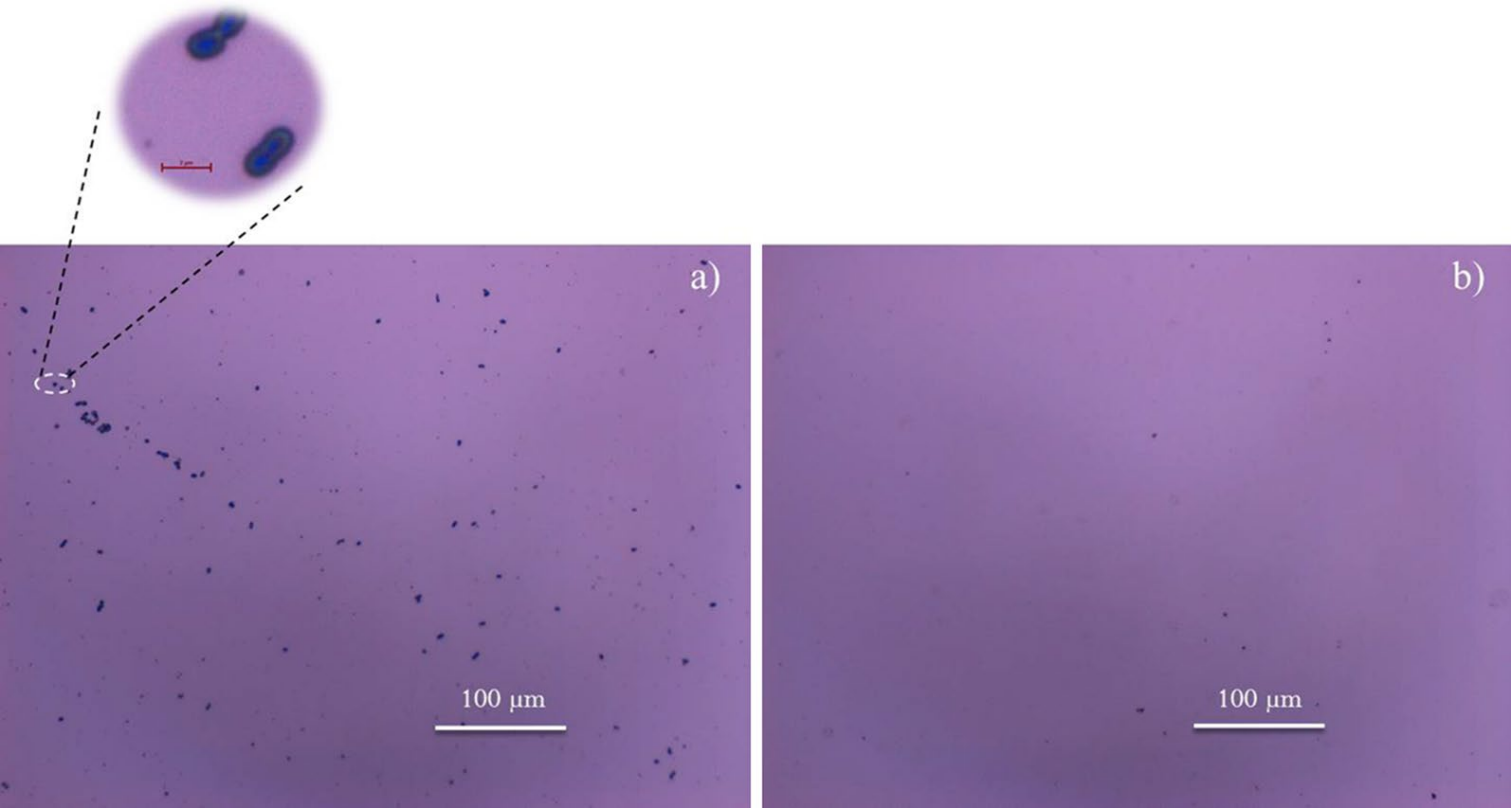
Representative microscope images of Piranha solution cleaned (**a**) and PFDT molecules modified silicon surface (**b**) after immersion test in *E. coli* culture for 24 h. The population of *E. coli* cells attached on the Piranha solution cleaned silicon surface is much higher than that on the PFDT molecules modified silicon surface. We can therefore conclude that the PFDT coated silicon surface possesses fouling resistant properties regarding *E. coli* (*E. coli* cells were stained by methylene blue)

**Fig. 4:**
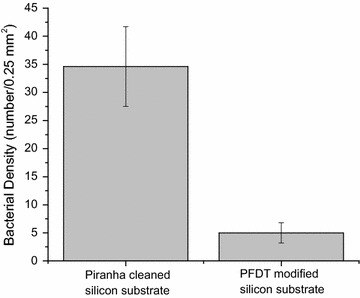
Attachment of *E. coli* cells on Piranha solution cleaned and PFDT coated silicon surface

Similar to the results obtained with *B. braunii*, *E. coli* cells readily adhere on Piranha solution cleaned silicon substrate as well (Fig. 3a). After PFDT modification, a significant reduction in the number of adherent *E. coli* cells was observed (Fig. 3b), confirming the antibacterial efficiency of the PFDT modified silicon substrate. Attached bacterial densities were calculated (Fig. 4). Five fields of view (0.25 mm^2^) on five replicate substrates were analyzed for each surface condition.

**Fig. 5:**
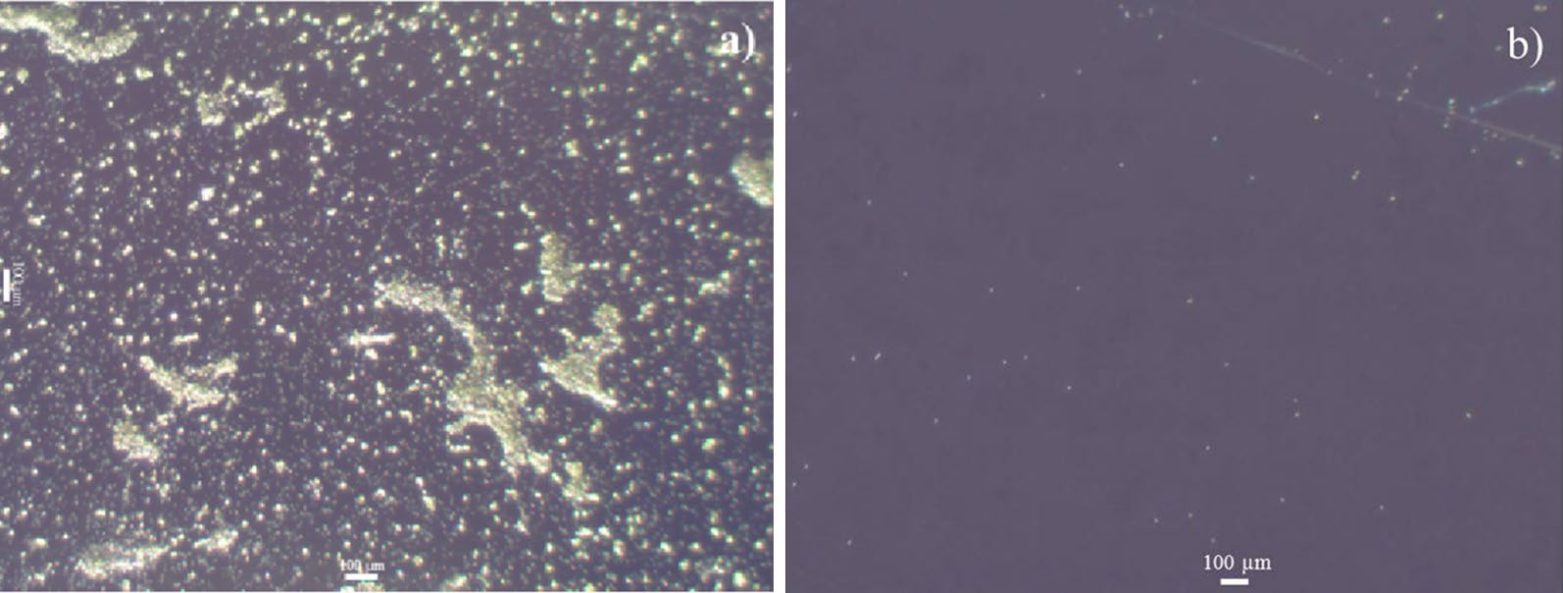
Representative microscope images of PFDT molecules modified silicon surface after immersion test in *B. braunii* culture for 1 month at room temperature (**a**) and such surface after immersing in Bristol medium containing 10 mM TCEP·HCl for 1 h at room temperature (**b**)

Figure 5a shows the micrograph of the PFDT modified silicon surface after 1 month of incubation in a *B. braunii* culture. As can be seen in the micrograph, the cell density, the percentage, and the average area of spread of *B. braunii* cells increased significantly throughout the test, indicating the reduced antifouling performance of PFDT modified silicon substrate. Our experimental results are in accordance with previous reports that pre-microbial attachment can provide specific binding sites for further attachment of microbes and growth. The *B. braunii* microcolonies formed on the PFDT modified silicon substrate during a 1-week immersion test in order to undergo further adaption and development into *B. braunii* macrocolonies. However, when the sample was incubated in Bristol medium containing 10 mM TCEP·HCl for 1 h at room temperature followed by rinsing with Bristol medium, the cell density decreased noticeably. It is because the reducing agent, TCEP·HCl, released the PFDT layer through breaking the disulfide bond, and therefore detached the algal cells from the surface. Although several *B. braunii* microcolonies remained on the surface after TCEP·HCl treatment, it might have been due to the uneven coverage of the PFDT coating. The oxidation reduced the amount of thiol groups on the surface that could graft PFDT molecules via disulfide bonds. The attachment of *B. braunii* cells to the parts where no PFDT molecules were grafted could not be interrupted by TCEP·HCl.

**Fig. 6:**
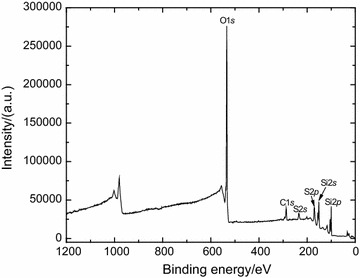
XPS survey spectrum of PFDT molecules modified silicon surface after incubating in 10 mM TCEP·HCl solution for 1 h

**Fig. 7:**
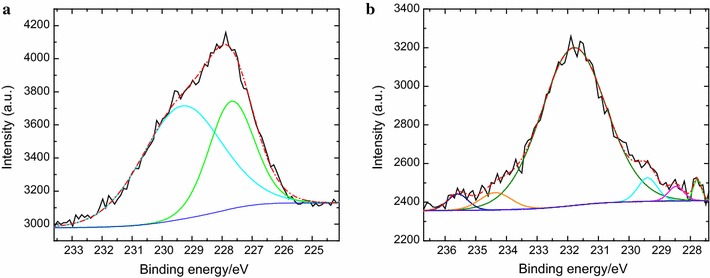
High-resolution XPS spectra of S 2*s* region of a freshly prepared thiol-terminated silicon surface (**a**) and a PFDT coated silicon surface after TCEP·HCl treatment (**b**). The black line is the raw data; colored lines denote individual fit components

As a control experiment, a PFDT modified silicon substrate without *B. braunii* immersion test was submerged in 10 mM TCEP·HCl solution for 1 h followed by rinsing with DI water, and then dried under a stream of N_2_ (g) at room temperature. The lack of F peak in XPS survey spectra (Fig. 6) confirms that PFDT was detached from the surface after TCEP·HCl solution treatment. Peak-fitting of the S 2*s* envelope was utilized to analyze a change in the chemical state of terminal sulfur on the silicon surface. The S 2*s* peak instead of the S 2*p* peak was used in this analysis because the S 2*p* peak could avoid any possible overlap of the S 2*p* (160–169 eV) region with the Si 2*s* (155–165 eV) signal from the substrate [19]. It also because S 2*s* appears as a simpler, single peak, and not a spin–orbit doublet as does S 2*p* [20]. It was reported that the peak at nearly (227.6 ± 0.1) eV was assigned to the thiol group [20]. Based on the peak areas from the deconvolution exercise (Fig. 7), thiol groups decreased from 35.3% of the surface-bound sulfur (Fig. 7a) to 1.6% (Fig. 7b). This result indicates that thiol groups released from disulfide bonds (–S–S–) through TCEP·HCl treatment were oxidized to a significant extent.

**Fig. 8:**
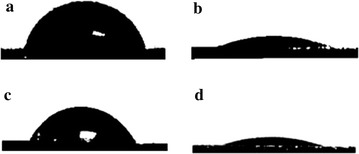
Water contact angle profiles captured on hydrogen-terminated (**a**), thiol-terminated (**b**), PFDT molecules modified (**c**), and PFDT molecules modified with TCEP·HCl treated (**d**) silicon substrates

Besides XPS, the difference in the chemical compositions was also reflected in the surface wettability of a given sample. The representative water contact angle images of chemically-modified silicon substrates in different stages are shown in Fig. 8. For the freshly prepared hydrogen-terminated silicon substrate (Fig. 8a), the water contact angle was about 78.4 ± 1.1°. After thiolation, the water contact angle was about 23.6 ± 1.2° (Fig. 8b). The surface wettability transformation from 78.4 ± 1.1° to 23.6 ± 1.2° can be attributed to the hydrogen bonds formed between thiol groups on thiolated silicon substrate and water molecules. After chemical modification with PFDT, the water contact angle was changed to about 70.0 ± 1.9°. This can be attributed to the introduction of the low surface energy of PFDT. After the TCEP·HCl treatment, the water contact angle was changed to about 22.1 ± 1.6° (Fig. 8d), implying the release of the PFDT layer from the surface.

**Fig. 9:**
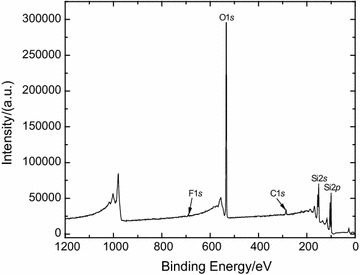
XPS survey spectrum of silicon surface prepared by first being modified with PFDT molecules, then treated with TCEP·HCl solution, and finally immersed in PFDT anhydrous ethanol solution for 2 h

Then, PFDT modified Si substrate with TCEP·HCl treatment was incubated in 100 mmol L^−1^ PFDT anhydrous ethanol solution again for 2 h. After being thoroughly rinsed with anhydrous ethanol by sonicating, the XPS result (Fig. 9) presents a F1*s* peak at 289 eV with weak intensity. This weak F1*s* peak indicates that the surface was barely modified by PFDT molecules which correspond to the loss of surface-bound thiol groups due to oxidation.

**Fig. 10:**
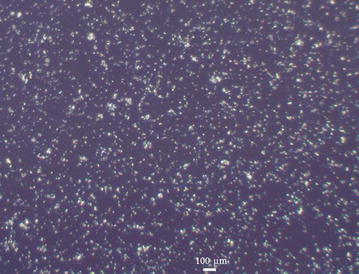
Representative microscope image of PFDT molecules modified silicon surface with TCEP·HCl solution treatment after immersion test in *B. braunii* culture for 1 week

The antifouling performance of the PFDT modified silicon substrate with TCEP·HCl treatment was investigated by following the procedures described previously. After 1 week of incubation in a *B. braunii* culture, the density of cells attached to the surface (PFDT modified silicon substrate with TCEP·HCl treatment) observed by microscope (Fig. 10) was greater than that on the PFDT modified silicon substrate without TCEP·HCl treatment. However, the cell density on the PFDT modified silicon substrate with TCEP·HCl treatment was lower than that on the Piranha solution cleaned silicon substrate. *B. braunii* cell clusters, which were formed on Piranha solution cleaned silicon substrate, were not observed on PFDT modified silicon substrate with TCEP·HCl treatment.

**Fig. 11:**
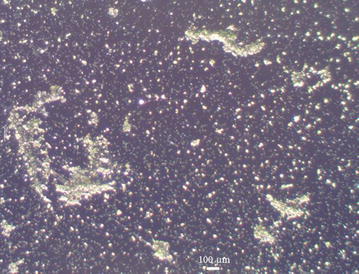
Representative microscope image of Piranha solution cleaned silicon surface which was first incubated in *B. braunii* culture at room temperature for 1 week, and then incubated in Bristol medium containing 10 mM TCEP·HCl at room temperature for 1 h

In order to evaluate the effect of TCEP·HCl on the attachment of *B. braunii*, a control experiment was conducted. A Piranha solution cleaned silicon substrate with *B. braunii* clusters (sample in Fig. 2a) was incubated into Bristol medium containing 10 mM TCEP·HCl for 1 h. From the microscope image (Fig. 11), the attachment of *B. braunii* is similar to that of Fig. 2a. This result indicates TCEP·HCl had no effect on the attachment of *B. braunii*.

## Conclusion

In summary, PFDT molecules were integrated onto thiol-terminated silicon substrate through the formation of disulfide bonds. The PFDT modified silicon substrate appeared to possess, to some extent, a micro-organism resistant property. However, as the time for the immersion test increased, the overall *B. braunii* cell density on the PFDT modified silicon substrate increased indicating its antifouling property cannot last forever. It was found that the adhered *B. braunii* on PFDT modified silicon substrate can be removed by applying TCEP·HCl solution. TCEP·HCl serves as a reducing reagent and can therefore break the disulfide bonds and detach the PFDT coating, along with the *B. braunii* cells adhered on it. This presented approach provides a rational design for removing antifouling coating that becomes aged, all without damaging the original substrate.

## Additional file

**Additional file 1: Figure S1.** XPS survey spectrum of PFDT molecules modified Si surface. **Figure S2.** Optical density of *B. braunii* culture at 660 nm.

## Abbreviations

PFDT: 1*H*,1*H*,2*H*,2*H*-perfluorodecanethiol
DMF: *N*,*N*-dimethylformamide
XPS: X-ray photoelectron spectroscopy
TCEP·HCl: tris(2-carboxyethyl) phosphine hydrochloride
LB: Luria–Bertani
*E. coli*: *Escherichia coli*
*B. braunii*: *Botryococcus braunii*
