# Supplementary material for: Surface thiolation of silicon for antifouling application
Source: Chem Cent J. 2018 Feb 7;12:10. doi: 10.1186/s13065-018-0385-6 (PMC5801134; doi:10.1186/s13065-018-0385-6)
Supplement: Supplementary file 1 — Additional file 1: Figure S1. XPS survey spectrum of PFDT molecules modified Si surface. Figure S2. Optical density of B. braunii culture at 660 nm. [file 13065_2018_385_MOESM1_ESM.docx]

**Additional Material**

**Figure S1.** XPS survey spectrum of PFDT molecules modified Si surface.

**Figure S2**. Optical density of *B.braunii* culture at 660 nm.
